# Landmark detection in 2D bioimages for geometric morphometrics: a multi-resolution tree-based approach

**DOI:** 10.1038/s41598-017-18993-5

**Published:** 2018-01-11

**Authors:** Rémy Vandaele, Jessica Aceto, Marc Muller, Frédérique Péronnet, Vincent Debat, Ching-Wei Wang, Cheng-Ta Huang, Sébastien Jodogne, Philippe Martinive, Pierre Geurts, Raphaël Marée

**Affiliations:** 1 Montefiore Institute, Department of Electrical engineering and Computer Science., University of Liège, Liège, 4000 Belgium; 2 Laboratory for Organogenesis and Regeneration, GIGA-Research, University of Liège, Liège, 4000 Belgium; 3 Institut de Biologie Paris-Seine (IBPS), UMR7622, Laboratoire de Biologie du Développement, UPMC Univ Paris 06, Paris, F-75005 France; 4 Institut de Systématique, Evolution, Biodiversité, ISYEB UMR 7205 (CNRS, MNHN, UPMC, EPHE), Muséum national d’Histoire naturelle, Sorbonne Universités, Paris, F-75005 France; 5 Graduate Institute of Biomedical Engineering, National Taiwan University of Science and Technology, Taipei, 10607 Taiwan; 6 Department of Medical Physics, University Hospital (CHU) of Liège, University of Liège, Liège, 4000 Belgium

## Abstract

The detection of anatomical landmarks in bioimages is a necessary but tedious step for geometric morphometrics studies in many research domains. We propose variants of a multi-resolution tree-based approach to speed-up the detection of landmarks in bioimages. We extensively evaluate our method variants on three different datasets (cephalometric, zebrafish, and drosophila images). We identify the key method parameters (notably the multi-resolution) and report results with respect to human ground truths and existing methods. Our method achieves recognition performances competitive with current existing approaches while being generic and fast. The algorithms are integrated in the open-source Cytomine software and we provide parameter configuration guidelines so that they can be easily exploited by end-users. Finally, datasets are readily available through a Cytomine server to foster future research.

## Introduction

Geometric morphometrics has become the dominant set of methods used to quantify size and shape of biological objects^1^. It involves the analysis of configurations of landmarks (i.e. discrete anatomical loci) among individuals and has been applied to a huge diversity of models and research questions, ranging from fossil humans^2^ or dinosaurs^3^ to butterfly wings^4^, zebrafish skeletogenesis^5^, or flower shapes^6^. Similarly, it can be applied in human medical imaging, e.g. cephalometry aims at analyzing human cranium for orthodontic diagnosis and treatment planning^7,8^. Typically, landmark positioning is first performed manually in individual two-dimensional images. Then, landmarks configurations are compared using, e.g., Procrustes superimposition^9^ and various multivariate statistics can be applied to characterize landmark configuration variations - and thus shape changes - in large populations. As such studies could typically involve hundreds or even thousands of individuals and tens of landmarks, the need to manually position the landmarks prior to such analysis is a very limiting factor. There is therefore a strong need for (semi-)automated landmark detection methods in biology.

In computer vision, the problem of landmark localization has been extensively studied in faces^10,11^. Methods for face analysis can however not be easily transposed to biological images, because of their very different and variable nature. The small size of ground-truth datasets typically available in biology also requires to design more data-efficient approaches. In biology, the landmark structure is also very different than in face images. Indeed, the number of landmarks of interest is typically small ($\sim 20$ landmarks) and the images typically large ($\sim 1500\times 1500$), which makes the landmarks more spaced apart than in face images. In the biomedical field, the problem of automatic landmark positioning has been addressed in cephalometry. Standard methods in this domain are based on the combination of template matching algorithms and prior knowledge information and differ mainly in their feature extraction steps (see *Kaur et al.*^12^ for a review). More recently, several successful landmark detection algorithms have been proposed in this domain that are based on pixel classification or regression using machine learning techniques followed by global landmark structure refinement^13–15^. Because these approaches have been proposed in the literature to tackle specific applications, such as face analysis or cephalometry, none of them was systematically evaluated on a broader range of biomedical applications.

In this paper, we study variants of a generic method for landmark detection that makes no specific assumption about the types of images to analyze and landmarks to detect. It is based on the extraction of multi-resolution features and the use of generic tree-based ensemble machine learning methods, namely (Extremely) Randomized Forests^16,17^. The choice of this latter family of machine learning tools is motivated by their many successful applications in computer vision in general and biomedical image analysis in particular^18^. Random decision forests have been exploited for example in various image classification tasks^19–21^, for organ segmentation in medical images^15,22^, but also for landmark detection in radiology^13–15^.

Our contributions in this paper are as follows:

- We propose a novel generic learning-based approach for landmark detection.
- We thoroughly study the effect of its parameters on three diverse bioimage datasets (for human cephalometric radiographs, zebrafish skeletogenesis, and Drosophila wing developmental studies). From this analysis, we derive guidelines for choosing these parameters on new problems.
- We compare our method with several landmark detection algorithms from the literature, both on the same three datasets and on two cephalometric challenges. These comparisons show that our approach yields competitive results in terms of accuracy, with lighter models and lower prediction times.
- We provide an open-source implementation of these algorithms through the Cytomine platform^23^ that further implements proofreading tools to combine automatic detection and manual refinements.
- As an important side contribution, we provide an easy access to the datasets used in this study with the hope that the landmark detection problem will gain more interest in bioimage informatics and machine learning research.

## Methods

### Materials

**Figure 1:**
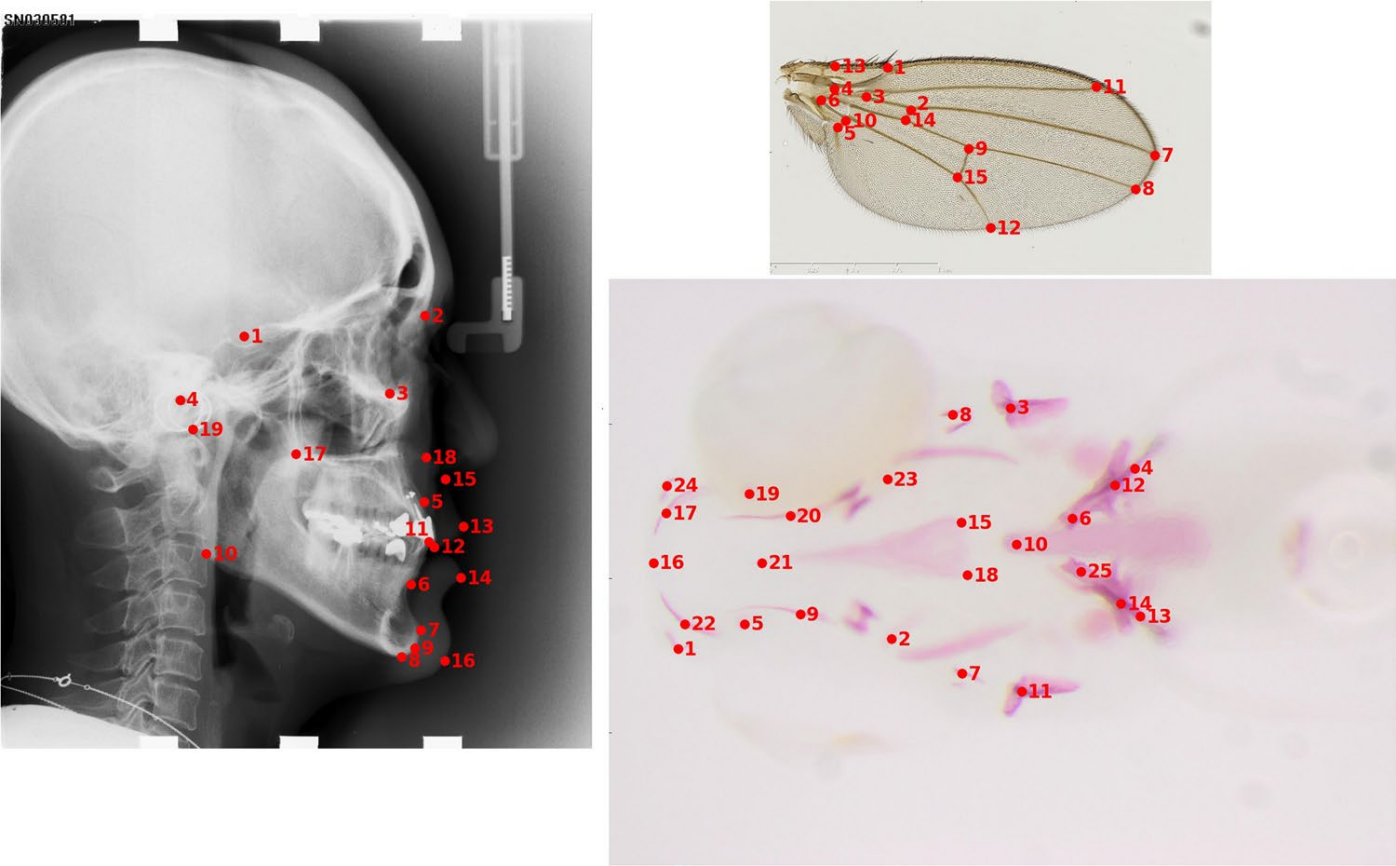
Sample image and corresponding landmarks for each dataset. CEPHA (left) with 19 landmarks, DROSO (top right) with 15 landmarks and ZEBRA (bottom right) with 25 landmarks.

We tested our method on three datasets summarized below. A more complete description is presented in the Supplementary Materials. An illustration of the landmarks is given in Fig. 1 with one image per dataset and their corresponding landmarks.

- **CEPHA**, a dataset of 100 lateral human cephalometric radiographs. This dataset has been previously described in *Wang et al*.^24,25^. 100 cephalometric X-ray images were collected from 100 patients aged from 6 to 60 years old. Image resolution is 1935 by 2400 pixels. 19 landmarks were manually marked and reviewed by two experienced medical doctors for each image. Additional images were provided during two international challenges^24,25^. Some of the landmarks corresponds to visual edges (7, 8, 13) while some others corresponds to morphological locations with less visual information (1, 4, 19).
- **DROSO**, a dataset of 138 colored images of Drosophila wings.
- **ZEBRA**, a dataset of 113 ventral views of head skeleton of zebrafish larvae. The image resolution is 2576 by 1932 pixels. 25 landmarks were manually marked and reviewed by the same expert. Most landmarks of this dataset corresponds to locations with little visual information (1, 2, 5, 15, 18, …).

### Algorithm description

We tackle the problem of landmark detection with a supervised learning approach, i.e., we exploit manual image annotations (i.e. (*x*, *y*) positions of each landmark in several images) to train recognition models for each landmark. These models are then used to predict each landmark position in new, unseen, images. Inspired by the work of *Stern et al*.^26^, we consider and compare two different learning methods for landmark detection: one based on pixel classification, and the other based on pixel distance regression. With the first one, a classification model is trained for each landmark separately to predict for each image pixel whether it corresponds to the position of the landmark or not. In the second method, a regression model is trained, also for each landmark separately, to predict for each image pixel its distance to the landmark. Regardless of the method (classification or regression), the models are trained from a learning sample composed of pixels extracted either in the close neighborhood of the landmark or at other randomly chosen positions within the training images. Notably, in this work, each pixel in the training sample is described by a vector of visual features at different resolutions. It allows to rely on local repeatable patterns and to disambiguate locally similar patterns using a wider context.

The different steps of the algorithm for a single landmark are explained in the following subsections. The whole procedure is repeated for each landmark separately.

#### Extraction and description of pixels

Each observation in the training sample corresponds to a pixel at some position (*x*,*y*) in one of the training images. Each pixel is labeled by a discrete or a numerical output, depending on the chosen method (classification or regression), and it is described by several input features. We list below successively the output associated to each pixel respectively in the classification and in the regression method, the input features used to describe them, and the pixel sampling mechanism.

**Classification output:** In principle, only one position in each image corresponds to the landmark, which means that if *N* training images are available, only *N* positive examples will be available to train our pixel classification model. To extend the set of positive examples, we consider as positive examples all pixels that are at a pixel distance at most *R* from the landmark, where *R* is a method parameter. More precisely, if the landmark is at position (*x*_*l*_, *y*_*l*_) in an image, then the output class of a pixel at position (*x*, *y*) in the same image will be 1 if (*x* − *x*_*l*_)^2^ + (*y* − *y*_*l*_)^2^ ≤ *R*^2^, 0 otherwise.

**Regression output:** With the regression method, the output associated to each pixel is the euclidean distance between this pixel and the landmark position in the training image. If the landmark is at position (*x*_*l*_, *y*_*l*_) in an image, then the output value of a pixel at position (*x*, *y*) in the same image will be:

1 $$\sqrt{{(x-{x}_{l})}^{2}+{(y-{y}_{l})}^{2}}$$

**Multi-resolution input features:** In contrast to current algorithms where single-resolution features are extracted^14,15,26^, in this work, we capture the context of the landmark at different scales and distances. A pixel at location (*x*, *y*) will be described by D multi-resolution square windows of resized height and width 2*W* + 1 centered at its position (*x*, *y*), where *W* is a method parameter.

To this goal, images are downsized to *D* different resolutions prior to the windows extraction and the *D* resulting feature vectors are concatenated. For our images of size *m* × *n* pixels, these resolutions will be:

2 $$\frac{m}{{2}^{i}}\times \frac{n}{{2}^{i}}\forall i\in [\kern-2pt[ \mathrm{0..}D]\kern-2pt] $$

**Figure 2:**
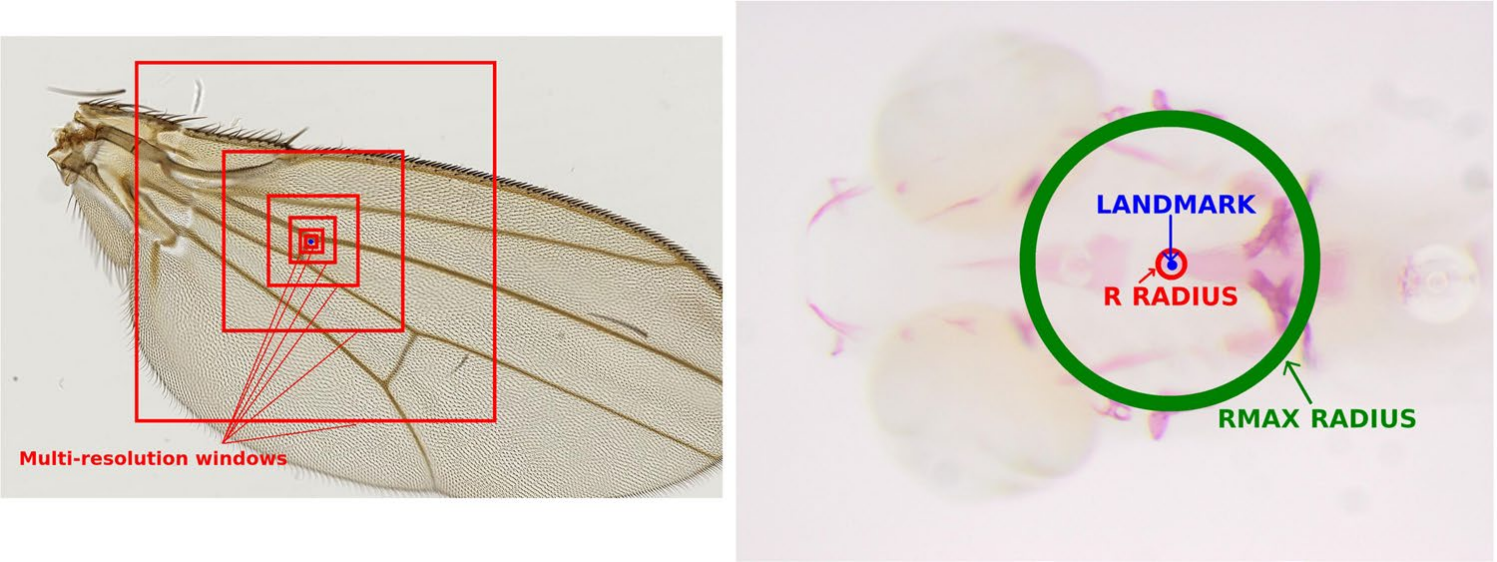
On the left, illustration of multi-resolution features representing one pixel (on the DROSO dataset, with *D* = 6 windows). The corresponding described pixel is located at the center of the windows (in blue). On the right, illustration of *R* and *R*_*max*_ radius (on the ZEBRA dataset). Observations in the *R* radius are considered as landmarks (positive) for the classification approach. At training, *PπR*^2^ non-landmark observations are extracted in the [*R*, *R*_*max*_] radius.

Out of image pixel values are set to zero. The influence of the chosen resolutions is shown in Section *Results*. An example of these windows is shown in Fig. 2.

We considered five ways of describing the multi-resolution windows:

- RAW: the raw pixel values of each resized windows are concatenated into a single vector. This will give a pixel descriptor of *D* × (2*W* + 1)^2^ features.
- SUB: the differences between the raw pixel values of the resized windows and the raw value of the pixel located at the (*x*, *y*) position. The pixel descriptor will thus also be of size *D* × (2*W* + 1)^2^.
- SURF: each window is described by the extended SURF descriptor^27^, a descriptor previously proven to show robustness against rotations, scaling and noise. The extended SURF descriptor consisting of 128 features, a pixel will be described by 128 × *D* features.
- GAUSSIAN SUB: the differences between the raw pixel values of *N*_*g*_ pixels and the raw pixel value of the pixel located at the (*x*, *y*) position on each of the *D* resolutions, where *N*_*g*_ is a method parameter. The *N*_*g*_ pixels are chosen according to offsets from the (*x*, *y*) position. These offsets are chosen randomly according to the gaussian distribution ${\mathbb{N}}\mathrm{(0,}\,\sigma )$, where *σ* is a user-defined parameter. In total, each pixel is represented by *D* × *N*_*g*_ features. Note that the window size *W* has no impact on this descriptor. Its role is taken by the parameter *σ* measuring the spread of the gaussian distribution.
- HAAR-LIKE: *N*_*h*_ Haar-Like features^28^ of random size and position are randomly extracted inside each of the *D* windows, leading to *N*_*h*_ × *D* features.

Gaussian sub features were proposed in *Donner et al*.^14^ and Haar-Like features were used in *Lindner et al*.^29^ to detect landmarks, in both cases however without multiple resolutions.

**Pixel sampling scheme at training:** Training a model on all pixels from all training images will be practically unfeasible in most cases and we will thus have to construct our training set by sampling the pixels. Uniformly sampling pixels from the training images will give however a very unbalanced learning problem for both classification and regression methods. For example, with a radius *R* = 20 pixels, only 1256 observations correspond, in classification, to positive examples, and in regression, to pixels within a distance <20 to the landmark. This is very small compared to the whole size of the images (e.g., about 4,000,000 pixels for our images). To generate a more balanced training sample, we select *π*(*R*)/(*s*)^2^ pixels within a radius *R* to the landmark in each training image, where *s* is a user-defined spacing parameter allowing to control the number of pixels extracted. *Pπ*(*R*)/(*s*)^2^ additional pixels are then randomly selected outside this radius, where *P* a user-defined parameter.

**Figure 3:**
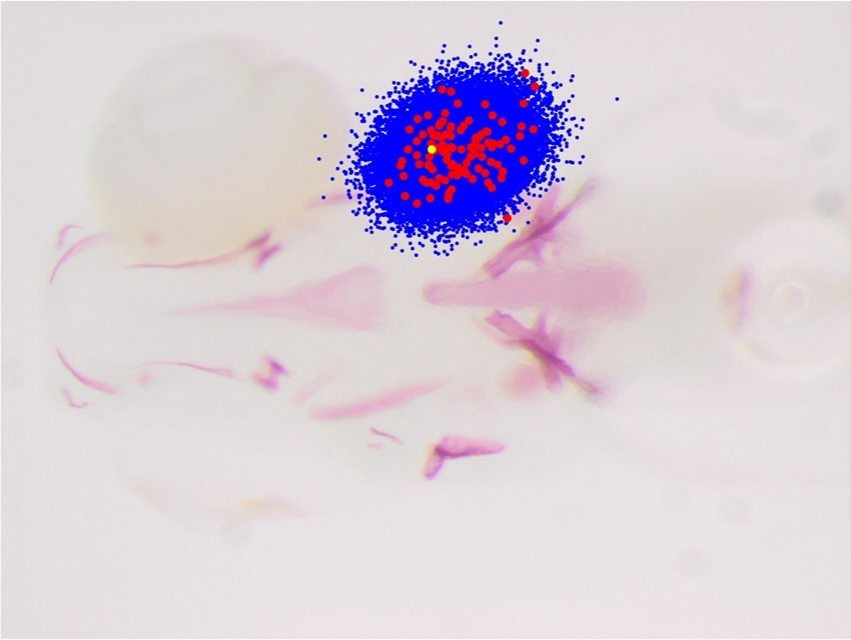
In red, the position of landmark 8 as observed in all the images of the ZEBRA dataset, overlaid on an image. In blue, the position of the corresponding 30,000 examples extracted during prediction according to our sampling strategy. In yellow, the real landmark position.

In practice, one can expect in many medical and biological applications that the same landmark will be located in close positions from one image to another (see Fig. 3 for an illustration on one of our datasets). At prediction stage, this information can be used to constrain the search for the landmark position to pixels that are not too far from the average position of the landmark in the training images. When this constraint is exploited at prediction stage, it is natural to avoid putting in the training sample pixels that are too far away from the landmark position. For this reason, we propose to select the *Pπ*(*R*)/(*s*)^2^ pixels outside the radius *R* uniformly at random within a radius *R*_*max*_ > *R* centered at the landmark position (see Fig. 2 for an illustration).

This subsampling contrasts with *Stern et al.*^26^ where pixels were sampled in the whole image during the training and the prediction phase.

**Robustness to rotations:** To improve robustness to rotations, and also to artificially increase the representativeness of the training data, we propose to expand our training set by adding artificially rotated versions of the training images. More precisely, to the original training set, we add *N*_*r*_ new versions of each training image, each obtained by rotating this image by an angle randomly selected between [−*α*, *α*], where *N*_*r*_ and *α* are two method parameters. With this operation, the total size of the training set will thus be multiplied by *N*_*r*_ + 1.

In the experiments, we will show that the problem of robustness to rotations is not important on our three datasets because the deformations stay small. On other datasets with bigger rotations, we would suggest to initially use a 2D registration algorithm such as presented in *Reddy et al*.^30^ in order to roughly align the images between each other before analysis.

#### Classification and regression model training

To train the pixel classifier or regressor, we will use the Extremely Randomized Trees algorithm^17^, a particular variant of Random Forests^16^. This method builds an ensemble of *T* fully developed decision or regression trees grown each from the original training sample (i.e., without bootstrapping). At each node, the best split is selected among *k* features chosen at random, where *k* can take its value between 1 and *m*, with *m* the total number of features. For each of the *k* (continuous) features, a discretization threshold is selected at random within the range of variation of that feature in the subset of observations in the current tree node. The score of each pair of feature and threshold is computed and the best pair among the *k* is chosen to split the node. As a score measure, we use Gini index reduction for classification and variance reduction for regression.

#### Landmark prediction

Let us denote by ${\mu }_{l}\in {{\mathbb{R}}}^{2}$ and ${{\rm{\Sigma }}}_{l}\in {{\mathbb{R}}}^{2\times 2}$ respectively the average and the covariance matrix of the landmark positions across the training images. To make prediction of the landmark position with our tree-based pixel classifier or regressor, we proceed as follows:

1. We randomly draw *N*_*p*_ pixel positions from a multivariate normal distribution ${\mathscr{N}}({\mu }_{l},{{\rm{\Sigma }}}_{l})$.
2. We apply the classification or regression model on each of the resulting pixels.
3. The predictions for these pixels are then aggregated as follows to obtain the final predicted landmark position:
  Classification: the final position is taken as the median position among the pixels that are predicted as being the landmark with the highest confidence by the tree-based model (i.e, every pixels which receives the highest number of votes for the positive class from the *T* trees in the ensemble).
  Regression: the final position is taken as the median position among the pixels that are predicted as being the closest pixels to the real landmark position (i.e, every pixels for which the predicted distance to the landmark position is the smallest).

The subsampling scheme of the first step is illustrated in Fig. 3. Such subsampling allows to improve predictive performance by reducing the probability of generating spurious landmark predictions at irrelevant positions in the images. It also considerably speeds up the algorithm with respect to an exhaustive scan of all image pixels as it was performed in *Stern et al*.^26^.

#### Implementation Details

Algorithms were implemented in Python using the scikit-learn library^31^ for its efficient implementation of the Extremely Randomized Trees algorithm and the OpenCV library^32^. All algorithms were further integrated into the open-source Cytomine web software^23^ using its software template mechanisms. End-users interested to interact with a Cytomine web-server through its web interfaces can read the Cytomine user guide (http://www.cytomine.be/Cytomine_userguide.pdf) and documentation (http://doc.cytomine.be), where complete instructions for using Cytomine and our landmark detection algorithms are given.

Most of the results presented in this paper were obtained on several computer clusters. About 20000 cluster jobs were needed for the complete cross validation of our algorithm, which represents about 10000 hours of computing time.

Note that on a regular computer (8 × 2.8 Ghz, 8 Go RAM), our implementation takes approximately one minute to build a model for a single landmark, and approximately one second to detect a single landmark on an image with *N*_*p*_ = 50000. A typical model (*R* = 15, *P* = 2, *T* = 100, *D* = 5) with ±100 images takes two gigabytes of RAM. Reducing the values of these parameters can lead to significant speed-ups, but it can also lead to a significant decrease of accuracy (see section *Results*).

## Results and Discussion

In this section, we will first study the behavior of our parameters through 10-fold cross validation. The goal of these experiments is to evaluate the influence of the method parameters and to use our three datasets in order to extract guidelines for their initial setting in future applications. We will then compare our results with existing algorithms. Finally, we will discuss the results and extract some guidelines for landmark detection on bioimage datasets.

### Influence of the method parameters

#### Experimental protocol

On each dataset, we used 10-fold cross validation to evaluate the performance for a given set of parameter values. We tested each parameter in turn, with all other parameters fixed to the default values given in Table 1 for each dataset. While analysing each parameter individually does not give information about the interactions that might exist between these parameters, the goal of our experiments here is mainly to identify the most influential parameters, ie., those that need to be carefully tuned for optimal performance. For optimal performance, these interactions need to be taken into account however by tuning these parameters jointly, using for example grid-search as in the comparisons of the next section.

**Table 1:** Description and default values of our method’s parameters at validation.

Parameter	Description	Default Value
*W*	The size of the multi-resolution window	8
*R*	The distance to the landmark position determining the training pixel output class	15 (CEPHA) 9 (DROSO) 20 (ZEBRA)
*s*	The spacing between the landmarks extracted inside the *R* radius	2
*R*_*max*_	the maximal distance to the interest point to extract non-landmark observations	600 (CEPHA) 300 (DROSO) 1000 (ZEBRA)
*P*	The ratio of negative versus positive examples sampled during training	1 (CEPHA) 2 (DROSO) 1 (ZEBRA)
*N*_*p*_	The number of pixels randomly extracted during prediction	30000
*N*_*r*_	The number of rotated versions of each training images that are introduced in the dataset	3
*α*	The maximal rotation angle (in degree)	30
*D*	The number of resolutions introduced in the feature representation of each window	5
*T*	The number of trees	50
*F*	The feature type used to describe the windows	RAW

In our experiments, we consider the euclidean distance of the predicted landmark position to the real landmark position as an error criterion. While other criteria have been proposed in the literature for specific applications^24^, the euclidean distance has the advantage to be applicable to any datasets and to be easily interpretable.

#### Results

Before discussing our findings, it is interesting to note that landmarks from the DROSO dataset are always detected with a significantly higher accuracy than landmarks from the two other datasets. This result is not surprising when looking at the sample images in Fig. 1. Indeed, landmarks from the DROSO dataset are clearly located at borders and intersections and are thus already easier to detect by human experts.

**Figure 4:**
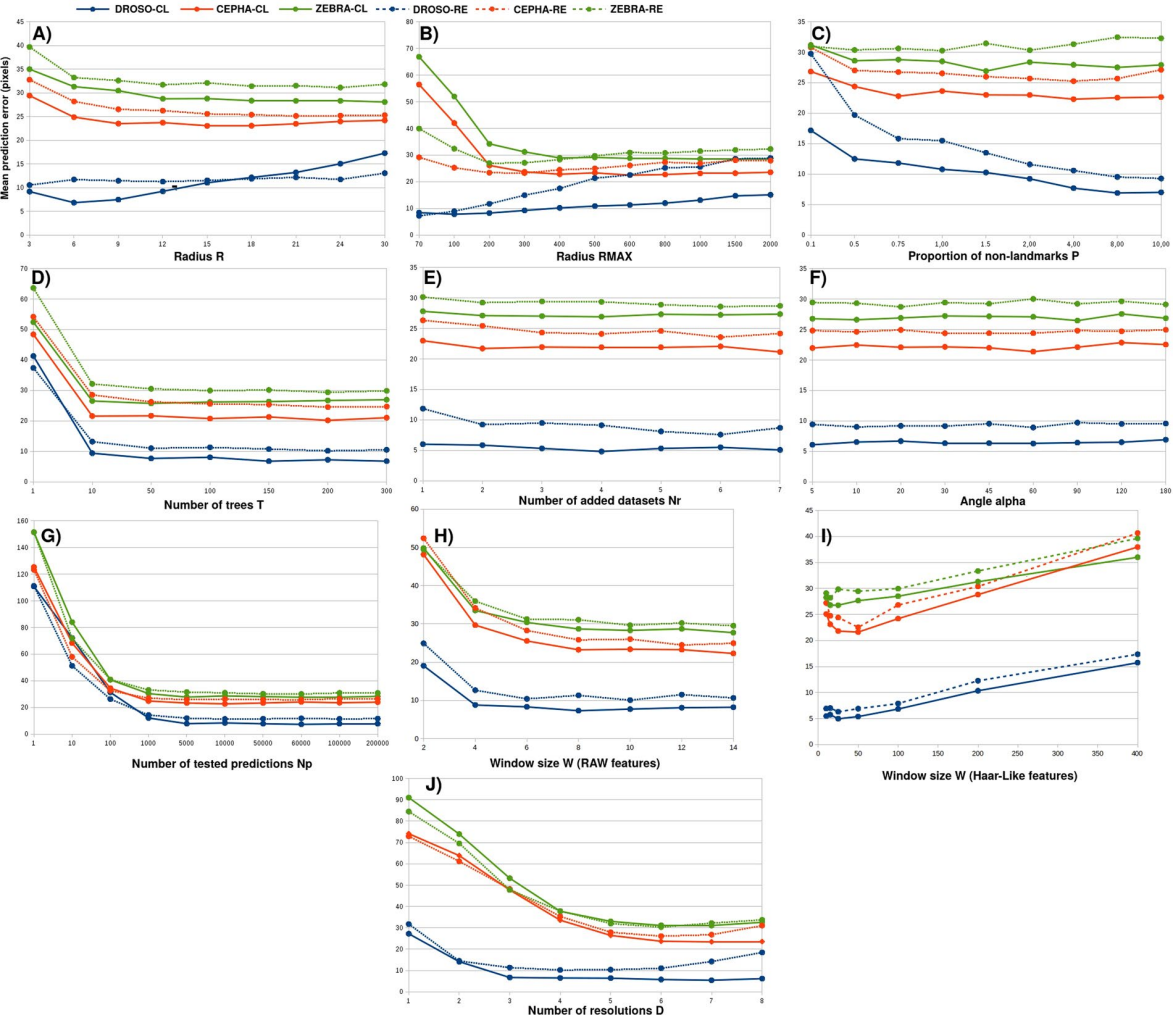
Influence of the parameters of our algorithm.

The influence of the **radius R** is presented in Fig. 4A. In **classification**, on CEPHA and ZEBRA, the higher *R* the better. On DROSO, where the landmarks are easy to detect, increasing *R* too much leads to a loss of accuracy. We explain this phenomenon by the fact that DROSO landmarks are intersections and edges, thus making the landmark position a highly informative position. Increasing *R* can thus only increase the confusion with close pixels. On the three datasets, a too small *R* has a negative impact on accuracy. This is probably due to a reduction in the number of the training examples. For CEPHA and ZEBRA, increasing *R* improves the accuracy. For these datasets, this increase allows to consider close well-defined structures and implicitly take into account the uncertainty on the exact location of the landmark (due to variation with the manual annotations by human experts). The radius *R* has less impact in **regression** than in classification although the main trends are similar. Note that the regression approach is expected to be less impacted by *R* because of its continuous outputs while this directly affects the binary output in classification.

The influence of the **radius** *R*_*max*_ is presented in Fig. 4B. In **classification**, we noticed quite similar effects on the three datasets: a small *R*_*max*_ can lead to confusion at prediction time with pixels far from the landmark, while a large *R*_*max*_ increases the probability of confusion with close pixels, because there will be proportionally less pixels close to the landmark in the training set. On ZEBRA and CEPHA, large values of *R*_*max*_ work better, suggesting that there is more confusion with remote pixels. On DROSO, smaller values are preferable, suggesting there is more confusion with closer pixels. This parameter thus needs careful tuning although *R*_*max*_ ∈ [200,300] seems to bring results close to the optimum. The main trends are similar in **regression**, however in this case the error clearly increases on all three datasets when *R*_*max*_ is increased too much. This might be explained by the fact that increasing *R*_*max*_ directly increases the range of the output values (i.e., distances) considered during model training. This could have a negative impact on the prediction error for small distances that are the only predicted distances used to determine the final position of the landmark.

The influence of the **ratio** *P* is presented in Fig. 4C. In **classification**, increasing the ratio *P* of negative versus positive examples significantly improves the results on the DROSO dataset, while, even if it is positive, the impact is more subtle on CEPHA and ZEBRA. Actually, looking at the same curves for individual landmarks (results not shown), we notice that increasing *P* has a negative impact for some landmarks on these two datasets. On DROSO, because landmarks are easier to detect, increasing *P* will decrease the risk of confusion with pixels outside the *R* radius. On CEPHA and ZEBRA, giving more weights to pixels outside the radius *R* increases the chance not to detect as positive pixels inside the radius, which has a negative impact on accuracy. The trends are very similar in **regression**: on DROSO, the larger *P*, the better, while on CEPHA and ZEBRA, the optimum value is landmark dependent.

The influence of the **number of trees** *T* is presented in Fig. 4D. For both **classification** and **regression**, the impact of increasing the number of trees is always positive as expected. We can observe however that increasing the number of trees beyond 50 does not bring improvement.

The influences of the **number of rotations** *N*_*r*_ and the **maximum value of the angle** *α* are presented in Fig. 4E and F. The rotations do not seem to have an impact on the (average) error. This suggests that our pixel descriptors are robust enough to orientation changes in our three datasets. Additional information about the robustness of our pixel descriptors to rotations is supplied as Supplementary Material.

The influence of the **number** *N*_*p*_ **of pixels tested at prediction** is presented in Fig. 4G. On our three datasets, we observe that *N*_*p*_ should be at least 10,000 to reach convergence. This number is equivalent to a complete search in a window of size 100 × 100 (i.e respectively 0.77,0.21 and 0.2 of the full images on the DROSO, CEPHA and ZEBRA datasets). Increasing *N*_*p*_ beyond 10,000 does not significantly improve the results.

The influence of the **window size** *W* is presented in Fig. 4H and I respectively for RAW and HAAR-LIKE features. For this parameter, we made a distinction between RAW and HAAR-LIKE features, because the size of *W* does not influence the size of the feature vector when using the latter type of features. Also note that when using GAUSSIAN SUB features, the algorithm is not influenced by this parameter. When using RAW features, increasing *W* up to 8 seems to significantly improve the results for the classification and regression approaches. Then, the marginal improvement decreases and, for DROSO, becomes negative. While small windows do not contain enough information, larger ones could create an overfitting problem due to the quadratic increase in the number of features. Because this quadratic increase will also affect the computational cost of our problem, we did not consider higher values. When using HAAR-LIKE features, the size of *W* still needs to be kept small, higher resolution information being provided by the use of multi-resolution windows. Optimal *W* values are in this case included between 15 and 20. This experiment was performed using the same number of descriptors per pixels as when considering RAW feature descriptors (1536).

**Figure 5:**
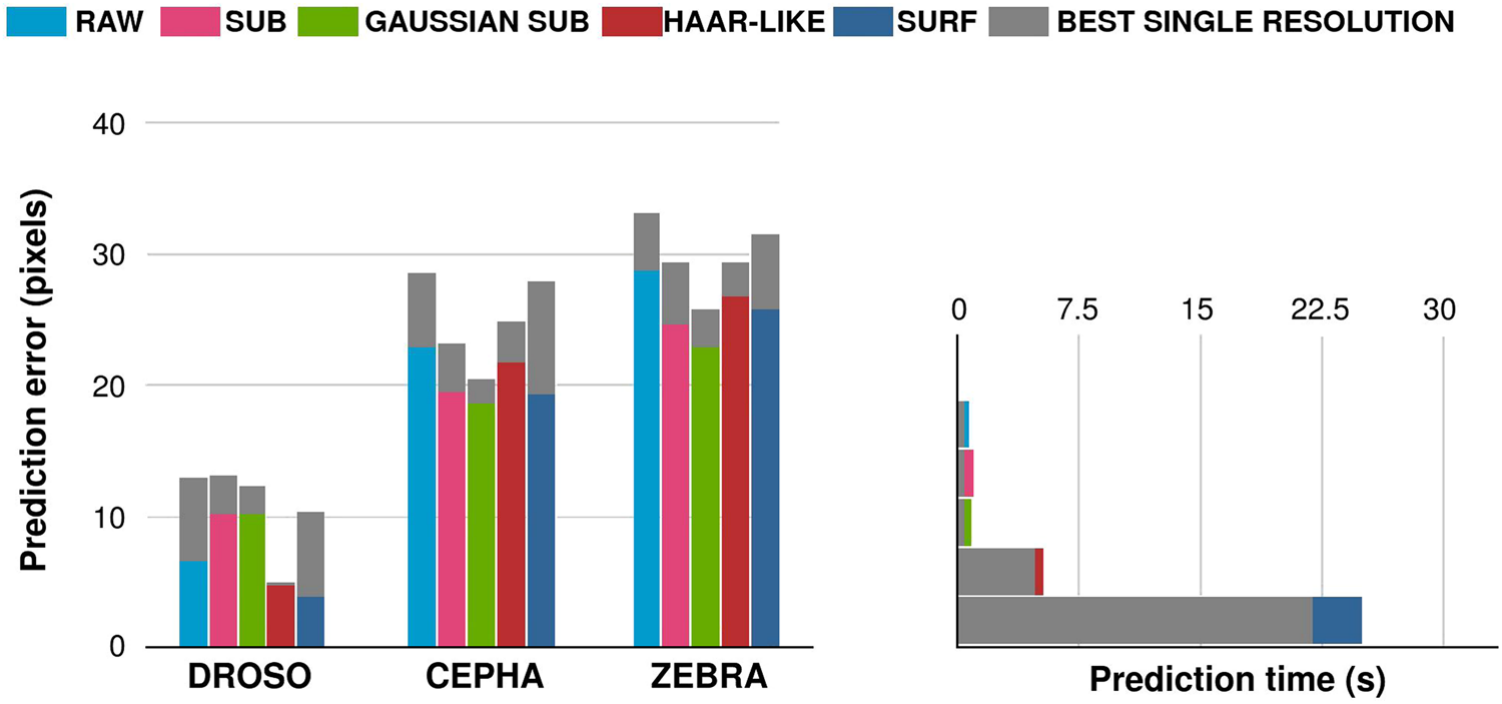
Influence of the use of the window descriptor features using classification. Above: the mean error using each window descriptor on each dataset. Below: the time needed for extracting 10,000 observations using each window descriptor. Grey bars represent the results obtained by using only the best resolution found during a 10-cross validation process.

The influence of the **window descriptor features F** is analysed in Fig. 5. In this figure, we also added single resolution features **SR** in order to analyze the influence of our multi-resolution approach. The best resolution was validated among the six resolutions used by our pixel descriptor. The *σ* of GAUSSIAN SUB (SR) was validated among six values (10, 25, 50, 100, 200, 400). The *W* of HAAR-LIKE (SR) was validated among six values (8, 20, 50, 100, 200, 400) and the *W* parameters used with other features was also validated among six values (8, 15, 20, 25, 30, 40). The total number of features for HAAR-LIKE (SR) and GAUSSIAN SUB (SR) was set to be the same as the number of descriptors for RAW and SUB (1536 descriptors).

From Fig. 5, we can conclude that using multi-resolution features always improves the performances while only slightly increasing the prediction time (it takes about one second for the resizings of a 2576 × 1932 image in our python implementation when *D* = 5).

Subtracting the value of the central pixel (SUB, GAUSSIAN SUB) instead of using raw pixel (RAW) values has a positive impact on both CEPHA and ZEBRA, while it clearly has a negative impact on DROSO. This is most probably due to the particular nature of the landmarks on the DROSO dataset. Landmarks in this dataset mostly correspond to borders and intersections that are rendered more difficult to detect when centering the pixel values. The SURF descriptor always performs well when using multi-resolution windows, but is only significantly better on the DROSO dataset. The GAUSSIAN SUB descriptor obtains the worst results on the DROSO dataset, but the best on the CEPHA and ZEBRA datasets. This suggests this feature descriptor is only efficient for landmarks located in areas with low visual information. The opposite goes for the HAAR-LIKE pixel descriptor, which is the most efficient on the DROSO dataset.

From the results, we can also conclude that while RAW and SUB do not obtain the best results on any of the datasets, these basic descriptors always obtain good performances. This is interesting when considering GAUSSIAN SUB and HAAR-LIKE: while they obtain the best performances on a given dataset, they obtain the worse for another. Given their small prediction time, we thus think RAW and SUB can be used to reach good performances on any dataset.

The influence of the **number of resolutions** *D* is presented in Fig. 4. In both **classification** and **regression** and for all datasets, increasing *D* first leads to a strong reduction of the error. At some point however, the error starts increasing. In average over all landmarks, the best performance is obtained by using *D* = 6 on our datasets.

### Comparison with other algorithms

In this section, we compare the results obtained by our method with several landmark detection algorithms. Because there is no available implementation of these algorithms, these comparisons are divided in two parts. First, we compare our method with our own implementation of the algorithms presented in *Donner et al*.^14^ and *Lindner and Cootes*^15^ on our three datasets, using only the original number of landmarks. Second, we present the results obtained by all algorithms on two cephalometry datasets used during international landmark detection challenges^24,25^.

#### Comparisons on our datasets

We compared our algorithm with our own implementations of the algorithms presented in *Donner et al*.^14^ and *Lindner and Cootes*^15^. The algorithm presented in *Lindner and Cootes*^15^ (called **LC** in the rest of the paper) is divided in two phases: first, individual landmark offset regressors are trained. These landmark offset regressors are then used to build vote maps giving the likeliest position of the landmarks. In a second step, these vote maps are combined with a PCA based model of the landmark shapes in order to build an active shape model for which an optimal configuration can be found through an iterative process. In a related study, they further improved the method performances on cephalometric data by considering a larger dataset and evaluating the accuracy of two different experts annotating the images^7^. In Donner *et al*.’s paper^14^ (called **DMBL** in the rest of the paper), a three step approach is proposed: first, a random forest classifier is trained to classify pixels. For *N* landmarks, the classifier associates to each pixel one class among *N* + 1 classes: either the type of a landmark or the background. This first step will be used to build a probability map for each of the landmarks. These probability maps will be refined into a small number of candidates for each landmark by using landmark offset regressors. The final position of the landmarks will be chosen among the candidates of each landmark using a Markov Random Field based on the distances between the landmark positions.

We estimated the results obtained with these two methods and ours on each dataset and landmark. We divided our datasets into a training and a test set. For each dataset, the methods were tuned on the training set using 10-fold cross validation. The models were then built using the complete training set and then evaluated on the test set. We chose to use half of the dataset images as learning set, and the other half as test set. For parameter exploration during CV, we used a grid search where some common parameters were fixed for a fair comparison: the number of trees, used in all methods, was fixed to 50. The number of descriptors for a pixel was also roughly fixed: our algorithm used 1536 features while LC and DMBL were set to use 1600. The tested values are presented in Table S1 of the Supplementary Material. The algorithms were all implemented in Python.

**Figure 6:**
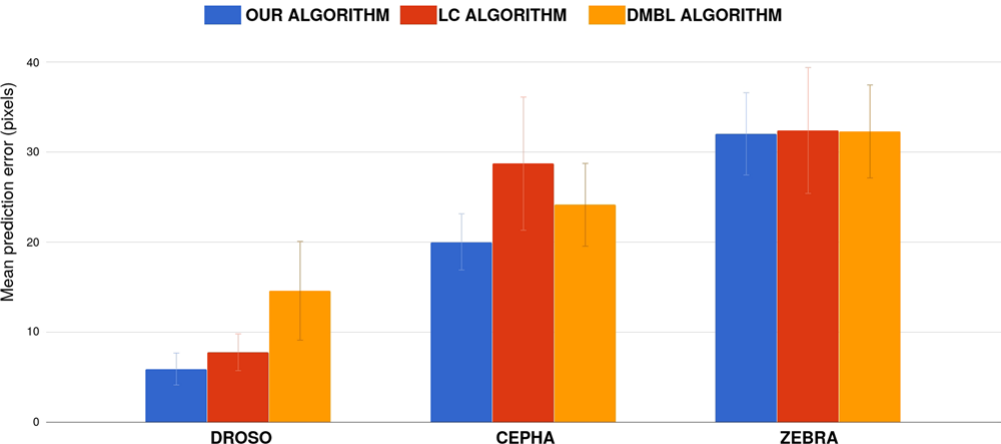
Comparison of our algorithm with *Lindner et al.*^15^ (LC) and *Donner et al.*^14^ (DMBL) on our three datasets. Error bars corresponds to 95%.

The results are presented in Fig. 6. We obtained the best results on each of the three datasets. On the DROSO dataset, that seems to be the easiest for all the algorithms, DMBL performs clearly worse than our algorithm and LC. We explain this by the feature engineering choice made in DMBL, whose pixel descriptors focus less on local appearance. On DROSO, our algorithm has only a small advantage compared to LC. However, our algorithm obtains the best performances for 10 landmarks, LC 5, and DMBL 0. On the CEPHA dataset, LC seems to obtain worse results than our method and DMBL. From our observations, we suspect that our algorithm performs better due to unreliable landmark candidates for DMBL and the correction phase of LC, that imposes too much constraints on the possible shapes described by the landmar. On the ZEBRA dataset, the results of the three algorithms are really close, but our algorithm obtains the best averaged error. Our algorithm obtained the best performances for 12 landmarks, LC 6, and DMBL 7. We also suspect that the landmark structures are not defined well enough when using small training image datasets (69 images for DROSO, 50 for CEPHA and 57 for ZEBRA), thus making the refinement steps of LC and DMBL less useful.

#### Comparisons on the ISBI cephalometry challenges

We considered a comparison with the preliminary version of our algorithm that was presented in the context of the 2014 ISBI challenge^24,33^. The goal of this challenge was to predict the position of each of the 19 cephalometric landmarks presented in Fig. 1 as accurately as possible. Here we focus on the comparison of our algorithm with the best results obtained during these two challenges. For the 2014 challenge, we compared our algorithm with its preliminary version that ranked second at the challenge, and with the challenge best performer, a method proposed by *Ibragimov et al*.^13^ (called **ILPV** in the rest of the paper). ILPV combines random forests with game-theoretic tools that take into account relations between landmark positions. To improve their performance at the challenge, ILPV furthermore manually created new landmarks that they incorporated in their training phase. The main difference between our preliminary and proposed method are the parametrization of the sampling at training and prediction, the use of rotations and the possibility to use SUB, HAAR-LIKE and GAUSSIAN SUB descriptors. We also compared our method with the 2015 ISBI challenge two best performers: ILPV that ranked second, and LC that ranked first. In the context of this challenge, they also used 10 additional manually annotated landmarks in order to ensure the consistency of their active shape model optimization problem. Note that LC is one of the state-of-the-art algorithms we tested in the previous section (but without any addition of manual landmark annotations).

**Figure 7:**
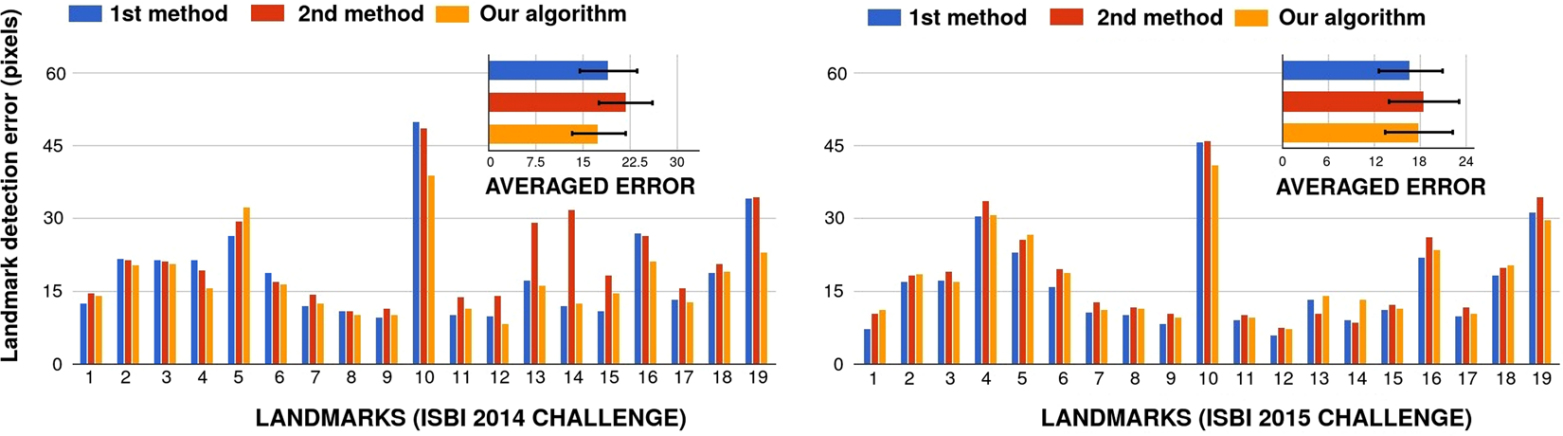
Comparison with 2014 and 2015 ISBI Cephalometric X-Ray Challenge best results.

The landmark by landmark comparison between the three methods for both challenges is presented in Fig. 7. This comparison was performed according to the challenge rules: we used the initial training sets (different for each challenge) composed of 100 images for the 2014 challenge and 150 images for the 2015 challenge. We tuned the parameters by 10-fold internal CV on the training set, trained the final models with the optimal parameters found on the whole training set, then compared the predictions of the different methods on the test set composed of 100 new images for both challenges. For parameter tuning, we used the same grid search as in the previous section. For the first challenge, the global mean accuracy of the preliminary version of our algorithm is 21.84 pixels. ILPV obtains 18.989 and the method proposed in this paper 17.55. For most of the landmarks, we observed a clear improvement between the previous and the novel variants of our algorithm. This shows that in some cases, using multi-resolution raw pixel values can help to obtain a better accuracy. On 11 out of 19 landmarks, our method works also better than ILPV, while it is outperformed on 8 landmarks. Given ILPV requires some additional manual annotation effort to reach this performance, we believe that our results are very competitive. For the second challenge, the global mean accuracy of the first method (LC) is 16.74 pixels and the second (ILPV) 18.46 pixels while we obtained a mean accuracy of 17.79 pixels. We obtained the best results for 3 landmarks, the second best results for 10 other landmarks. As we show in Fig. 7, it is clear that the differences are also small between the different methods. In comparison to these two other methods using additional landmarks to refine the landmarks shape, we think our algorithm, without additional annotation and refinement, is competitive with the state of the art.

#### Comparison of speed and memory consumption

Table 2 compares the four algorithms in terms of training and prediction speed as well as memory occupation. In terms of **memory occupation**, at **training**, our algorithm will build *N* classification or regression mono-output models. These models will be built by extracting around *N* different datasets of pixels. LC will build *N* bi-output regression models as well as a PCA model. In our experiments, we extracted approximately the same number of pixels for this algorithm and ours. In DMBL, one classification model will be built for phase 1 and *N* bi-outputs regression models. In our experiments, each of these models were built using approximately the same number of pixels as in our algorithm. In addition, a Markov Random Field will be built. We did not reimplemented ILPV, but given its model features, we can reasonably estimate that a model would ask for approximately the same number of pixels than our algorithm. This means that, at training time, DMBL extracts the largest amount of pixels while the three other algorithms use approximately the same amount. At **prediction**, we showed we could already reach our best performances by extracting around 10,000 pixels per landmark, while the number of pixels to extract will depend on image size for LC, DMBL and ILPV. DMBL extracts height × width × *δ*^2^ pixels, where the optimal *δ* we found was 0.5. LC extracts (height × width)/(step^2^), and we found an optimal step of four. In the cephalometric challenge, ILPV extracts (height × width)/(step^2^), where step = 3 for the cephalometric challenge. In addition, DMBL will need to keep probability maps of size *N* × height × width × *δ*^2^, LC’s vote maps of maximal size *N* × (height × width)/(step^2^) and ILPV probability maps of size *N* × (height × width)/(step^2^). Given the size of our images and the variance of the landmark positions, our algorithm uses a significantly smaller amount of memory than other methods on our three datasets. Note however that with smaller images or more variable landmark positions, the memory requirement and time consumption of our method might exceed those of LC and ILPV. In terms of **speed**, at **training**, DMBL will need to extract slightly more pixels than the other algorithms, and build one additional forest. LC, DMBL and ILPV are building additional models based on the landmarks coordinates. This is why our algorithm can be considered as faster than the other algorithms at training. However, if parallelization capacities are available, all the models can be built at the same time, and the same goes for the pixel extraction. In this context, the bottleneck will be the difficulty of the pixel extraction, which will make LC, ILPV that both use single resolution Haar-Like features and/or our algorithm if we use SURF features the slowest algorithm. At **prediction**, given that we extract less pixels and do not use an additional refinement step, our algorithm should be the fastest as long as we are not using SURF features. Another bottleneck comes from the refinement step(s) because these steps can not be parallelized between each other. This is still playing in favor of our algorithm which has no refinement step.

**Table 2:** Comparison of the time and memory consumption of the algorithms. N is the number of landmarks.

	Our algorithm	Lindner *et al*.^7^	Donner *et al*.^14^	Ibragimov *et al*.^13^
**TRAINING**	**PIXEL EXTRACTION** N datasets - 1 dataset ± 100,000 pixels Extraction can be parallelized **MODEL BUILDING** N single output classification OR regression forests **ADDITIONAL OPERATIONS** Each training image must be resized D-1 time(s) If HAAR-LIKE features are used, integral images need to be built	**PIXEL EXTRACTION** N datasets - 1 dataset ± 100,000 pixels Extraction can be parallelized **MODEL BUILDING** N double-output regression forests 1 PCA model (using landmark coordinates) **ADDITIONAL OPERATIONS** Integral images need to be built	**PIXEL EXTRACTION** N + 1 datasets (1 phase 1 + N phase 2) - 1 dataset ± 100,000 pixels Extraction can be parallelized **MODEL BUILDING** 1 classification forest (N + 1 classes) N double output regression forests	**PIXEL EXTRACTION** N datasets - 1 dataset ± 100,000 pixels Extraction can be parallelized **MODEL BUILDING** N classification forests (2 classes) 1 gaussian model (fron coordinates) **ADDITIONAL OPERATIONS** Integral images need to be built
**LANDMARK DETECTION** (for 1 image)	**PIXEL EXTRACTION** *N*_*p*_ pixels - *N*_*p*_ << height × width (± 10–30,000) Extraction can be parallelized **ADDITIONAL OPERATIONS** Building of multi-resolution images Integral image (if HAAR-LIKE is used)	**PIXEL EXTRACTION** (height × width)/(step^2^) pixels Extraction can be parallelized **ADDITIONAL OPERATIONS** N vote maps are built Iterative active shape model optimization Integral image needs to be built	**PIXEL EXTRACTION** Phase 1: height × width × delta^2^ pixels (1–3 M) Phase 2: #Candidates × N × #iterations pixels (max.) (500–1000) Extraction can be parallelized (per phase) **ADDITIONAL OPERATIONS** N probability maps are built (size height × width × delta^2^) MRF optimization	**PIXEL EXTRACTION** height × width pixels Extraction can be parallelized **ADDITIONAL OPERATIONS** N probability maps are built (size height × width) GTF optimization Integral image needs to be built
**PIXEL DESCRIPTOR**	RAW, SUB, SURF, HAAR-LIKE GAUSSIAN SUB	HAAR-LIKE	GAUSSIAN SUB	HAAR-LIKE
**LEARNING MODEL**	Extremely randomized trees (classification OR regression)	Random forests (regression) PCA model	Extremely Randomized Trees (classification and regression) Markov Random Field	Random forests (classification) Gaussian model

### Guidelines

In this section, we provide guidelines to end-users to choose the best method and parameters for their applications.

#### Method choice

We showed that we could obtain good performances with our algorithm with respect to other landmark detection methods: while offering slightly better performances, our algorithm also creates lighter models and needs to extract less pixels during the prediction phase, thus offering a speed-up during prediction.

However, for datasets with a significantly higher number of landmarks (>40–50), we expect the algorithms of DMBL, LC and ILPV to bring better results than our own algorithm because they better exploit the global landmark structure for the localization of the landmarks. In this context, we would advise to use LC’s algorithm when landmarks are visually well defined locally, and DMBL otherwise.

Furthermore, our experiments showed that these algorithms could be improved by using some of our algorithm’s specificities:

- Different types of pixel descriptors should be considered: our experiments showed that the performances of a given pixel descriptor can vary from one dataset to another given the type of landmarks. Moreover, even if it slightly increases computation time, multi-resolution feature extraction improves the performances. In the context of a new application, we think that the choice of the pixel descriptor should always be empirically assessed, if not per landmark, at least per dataset.
- In most biomedical applications, the deformation between the images will remain small. We showed that this information can be used to reduce the number of pixels to extract at prediction time, and thus speed up the prediction process.

#### Parameter setup

Using our method, we recommend to use the classification approach as we showed it has a better accuracy in almost all settings compared to the regression approach. In addition, it is typically less demanding in terms of computing time and memory, mainly because it leads to significantly smaller trees.

According to our experiments, only *R*, *R*_*max*_, and *F* have to be tuned to optimize performance. The optimal value of these parameters are expected to be related to the appearance of the landmarks and they might thus be tuned for each landmark individually. On landmarks well defined at borders or intersections, small *R* values can be used (*R* = 6 for example). Given *R* and *R*_*max*_ are in pixel units, the range of variation of these parameters should also be adapted to the image resolution: at minimum, *R* must encompass the landmark possible locations, and *R*_*max*_ the nearest visible structures. We advise to use HAAR-LIKE features in the context of landmarks located at borders or intersections, and GAUSSIAN SUB features otherwise. If low computing time is a key factor, RAW and SUB features could also be assessed. Because it requires more computation time (on a factor 10) than RAW or SUB, we can only advise the use of the SURF descriptor for small datasets or given high parallelization capacities.

We also recommend to adapt the value of *N*_*p*_ to the variability of the position of the landmark in the training images. Indeed, given our sampling scheme at prediction, the more variable the position of the landmark, the more spread in the image will be the pixels tested to compute the final prediction. One should thus increase the number of tested pixels accordingly so as to ensure a fine enough coverage of the area where the landmark could be located. In our case, *N*_*p*_ = 10,000 seems a good option to start with.

Other parameters can either be set to some reasonable default value (*W* = 8,*D* = 6) or be set to their maximum value given the available computational budget (*T*, *P*, *N*_*p*_, *W*). If computational resources are plentiful, a finer tuning of *W* and *D* and *N*_*p*_ can bring some further improvement but according to our experiments this improvement is expected to be small in most cases.

We carried out our experiments on three very different problems, which gives some generality to the previous guidelines. Note however that on the three datasets, the deformations between the images are small, which for example allows to limit the region of interest within images where the landmark can be searched for. To cope with larger deformations, one could play with the value of some of the method parameters, e.g. increasing *N*_*r*_, and *N*_*p*_. However, in order to increase the performance and reduce computing times, we recommend either to control image acquisition to avoid large deformations or, if this is not possible, to perform rigid registration between the images before further analysis.

## Conclusion

Our results show that it is possible to accurately detect landmarks using a combination of randomized trees and pixel-based multi-resolution features. Given the small size of our datasets and the variance of the landmarks between the images, we think that these results are very satisfactory. The main advantages of our approach with respect to existing works are its efficiency, its independence to the number of landmarks, and its lower time and memory requirements. All evaluated algorithms are available through the open-source Cytomine platform^23^ (see Supplementary Material for a description), which provide proofreading tools so that end-users can actually speed-up their annotation processes by focusing on difficult landmarks.

In terms of future works, we think that improving accuracy on our three specific landmark detection tasks mostly require using more data: for some landmarks, ±100 images does not seem to be enough to grasp the variability of the possible landmark visual representations. Moreover, it seems that some landmarks do not especially correspond to specific anatomical locations, but more to geometric positions or intersections (e.g. landmark 10 on CEPHA dataset. See Fig. 1). This kind of landmark will thus naturally be incorrectly detected by our approach which exploits repeatable visual appearances without using global spatial information. We expect further accuracy improvement might be obtained by taking into account the relative positions and the global structure of the landmarks either directly during the training stage or as a post-processing during the prediction stage. Note that this structure is taken into account by current state of the art algorithms we compared ourselves with. However, given that their accuracy is similar to ours despite this post-processing, more research seems to be needed to find methods more adapted to our datasets. Further improvement might also be brought by adding some parametrization possibilities: for example, the windows could be fine tuned with different shapes at each of the different resolutions and *D* could be adapted to different ranges of image resolutions. Interestingly, as our datasets and source code are available through Cytomine^23^, we hope that other researchers will design new efficient landmark detection algorithms that can be plugged into the platform and compared with already implemented algorithms.

Finally, this algorithm can be extended and used as a first step to perform point-based 2D and 3D multimodal registration^34^, and also for geometric morphometrics in 3D^35,36^.

## Electronic supplementary material

Supplementary Information 
