# Supplementary material for: Landmark detection in 2D bioimages for geometric morphometrics: a multi-resolution tree-based approach
Source: Sci Rep. 2018 Jan 11;8:538. doi: 10.1038/s41598-017-18993-5 (PMC5765108; doi:10.1038/s41598-017-18993-5)
Supplement: Supplementary file 1 — Supplementary Information [file 41598_2017_18993_MOESM1_ESM.pdf]

Landmark detection in 2D bioimages for geometric morphometrics: a multi-resolution tree-based approach: Supplementary Material

Vandaele, R.[1] Aceto, J.[2] Muller, M.[2] Péronnet, F.[3]
Debat, V.[4] Wang, CW.[5] Huang, CT.[5] Jodogne, S.[6]
Martinive, P.[6] Geurts, P.[1] Marée, R.[1]

[1]Montefiore Institute, Department of Electrical engineering and Computer Science., University of Liège, Liège, Belgium
[2]Laboratory for Organogenesis and Regeneration, GIGA-Research, University of Liège, Liège, Belgium
[3]Laboratoire de Biologie du Développement, Institut de Biologie Paris-Seine, UPMC Univ Paris 06, Paris, France
[4]Institut de Systématique, Evolution, Biodiversité, Muséum national d'Histoire naturelle, Sorbonne Universités, Paris, France
[5]Graduate Institute of Biomedical Engineering, National Taiwan University of Science and Technology, Taipei, Taïwan
[6]Department of Medical Physics, University Hospital of Liège, University of Liège, Liège, Belgium

This supplementary material contains a detailed description of the datasets and the instructions to retrieve data, and source code. The third section of this document contains additional information and experiments evaluating the robustness of our algorithms.

1 Availability

1.1 Datasets

Datasets (images and ground-truth landmarks) are available online on a Cytomine web server [12] `http://www.demo.cytomine.be`. End-users can access data through Cytomine-WebUI using:

- login: **landmark**
- password: **dl$2015LA**

In order to retrieve the data automatically, a basic script located at `https://github.com/cytomine/Cytomine-python-datamining/tree/master/cytomine-applications/`

landmark_model_builder/download_datasets.py can be used. After the installation of the Cytomine python client (see http://doc.cytomine.be/x/TQK8), this basic script will use Cytomine RESTful API to retrieve the data using the following command:

```
$python download_datasets.py <repository>
```

The script will generate on the local filesystem one repository per dataset. Images will be located at repository/<number>.jpg, and the corresponding coordinate file will be located at repository/txt/<number>.txt. In the coordinate file, one landmark is described per line. The first number correspond to the x axis location, and the second number to the y axis location, in pixels. The origin of the coordinate system is located at the upper left of the image, and the down right corner of the image corresponds to width-1,height-1.

1.2 Source code

Algorithms are available on Cytomine-Python-Datamining github repository https://github.com/cytomine/Cytomine-python-datamining/tree/master/cytomine-applications/.

The source code for building the different models is available in the landmark_model_builder repository: build_[generic,lc,dmbl]_model.py contains the source code for our algorithm, LC and DMBL. The test_[generic,lc,dmbl].sh files can be used to set the algorithms parameters and build the models. The source code for predicting landmark positions on new images using the models built is available in the landmark_predict repository: landmark_[generic,lc,dmbl]_predict.py contains the source code for our algorithm, LC and DMBL. The test_[generic,lc,dmbl].sh files can be used to set the algorithms parameters and perform the detection. Please note that you will need to add the corresponding softwares to your Cytomine instance using the add_software files.

2 Dataset Description

In this Section we give more details about the context and describe materials and methods to obtain our images.

2.1 CEPHA

Cephalometry aims at analyzing human cranium for orthodontic diagnosis and treatment planning. This dataset has been previously described in [15, 14]. 300 cephalometric X-ray images were collected from 300 patients aged six to 60 years old. The images are acquired with Soredex CRANEX Excel Ceph machine (Tuusula, Finland) and Soredex SorCom software (3.1.5, version 2.0). Image resolution is 1935 by 2400 pixels in TIFF format. Regarding the ground truth data for evaluation, 19 landmarks were manually marked and reviewed by two experienced medical doctors for each image. An ethical approval was obtained to conduct the study with IRB Number 1-102-05-017, which was approved by the research ethics committee of the Tri-Service General Hospital in Taipei, Taiwan. The data is available in Cytomine Project LANDMARKS-NTUST-CEPHA.

2.2 DROSO

Developmental homeostasis enables the constancy of the phenotype despite genetic, environmental and stochastic variations. Precise quantification of morphological traits is paramount to estimate perturbations and tackle the genetic and molecular bases of developmental homeostasis. The Drosophila wing, with its plane and stereotyped structure, is well suited to quantify subtle variations in size and shape in a population that would reveal inefficient homeostasis. Fifteen morphological landmarks corresponding to intersections between veins or between veins and the margin are used to describe wing size and shape with geometric morphometrics [8].

w^{1118} flies were raised on standard yeast-cornmeal medium at 25°C. Crosses were performed between 5 females and 5 males and transferred each 48h. Thirty females from the total offspring were sampled and their wings mounted on one slide, dorsal side up, in Hoyer's medium. Slides were scanned with a Hamamatsu Nanozoomer Digital Slide scanner, running the Nanozoomer software with a 20x objective and an 8-bit camera. Wing pictures were separately exported into TIFF format using NDP.view with the 5x lens and oriented with hinges to the left (Images are 1440 by 900 pixels). The fifteen morphometric landmarks were manually acquired as described in [5]. The data is available in Cytomine Project LANDMARKS-UPMC-DROSO.

2.3 ZEBRA

The zebrafish is increasingly used for studying embryogenesis in vertebrates; its rapid development and the transparency of its embryos and larvae have led to the identification of several mutants deficient in skeletal morphogenesis [13]. In particular, the head skeleton is the first to undergo ossification, by first forming a cartilaginous matrix starting at 3dpf which is later converted into bone structures through perichondral ossification. Other bone elements are formed without a pre-existing cartilage matrix by endomembraneous ossification. These processes, and the precise and reproducible positioning of the different elements at different stages of development under normal conditions have been well studied and described [7, 3]. Many of the identified mutations affecting skeletogenesis [13] cause either absence or severe malformations of the different cartilage [13, 7, 3, 4, 9] or bone elements [11], however recent studies focus increasingly on the signaling pathways regulating the precise positioning and shaping of the different elements [2, 17]. For these studies, more precise, objective and quantitative methods for morphometric description of the head skeleton are required.

Here, Zebrafish (Danio rerio) were maintained under standard conditions [16] in the GIGA zebrafish facility (licence LA2610359). Rearing and breading were performed as previously described [1], all protocols for experiments were evaluated by the Institutional Animal Care and Use Committee of the University of Liège and approved under the file numbers 568, 1074, and 1264 (licence LA 1610002).

The calcified bone structures in the head skeleton of zebrafish larvae were stained using Alizarin red S (Sigma-Aldrich, Diegem, Belgium) as previously described [1]. Briefly, the larvae were fixed in 4% PFA for 2h at room temperature and rinsed several times with PBST (3.2 mM Na2HPO4, 0.5 mM KH2PO4, 1.3

mM KCl, 135 mM NaCl, 0.05% Tween® 20, pH 7.4), then pigmentation was bleached in a H2O2 solution (H2O2 3%, KOH 0.5%) and finally the larvae were rinsed 3 times in a solution of 25% glycerol / 0.1% KOH and 50% glycerol, 0.1% KOH. After the bleaching, long rinses (at least 20min each) in a 25% glycerol, 0.1% KOH solution are necessary to prevent fading of the staining. The larvae are stained in a 0.05% Alizarin red solution in water for 30min in the dark on low agitation, rinsed in a 50% glycerol, 0.1% KOH solution to remove excess staining and kept at 4°C in the same solution. Images of stained larvae (n=20-30 larvae) were obtained on a binocular (Olympus, cell B software) by placing the larvae in glycerol in a white plastic plate, using the same illumination and acquisition parameters for each session. Bitmap images are 2576 by 1932 pixels.

The data is available in Cytomine Project LANDMARKS-ULG-ZEBRA.

3 Experiments

3.1 Values tested during comparison

Table 1 shows the values that were tested during the comparison of the landmark detection algorithms. Full description of LC and DMBL parameters can be found in their corresponding papers [6, 10].

3.2 Robustness analysis

In this section, we will analyze the influence of the deformations in the images on the accuracy of our method.

We define the deformation of an image i, d_i as the euclidean distance between its landmarks and the mean shape (the mean position of the landmarks). This deformation is computed once the shapes have been centered:

$$\bar{x}_{i,l} = x_{i,l} - \frac{1}{L}\sum_{j=1}^{L} x_{i,j}, \bar{y}_{i,l} = y_{i,l} - \frac{1}{L}\sum_{j=1}^{L} y_{i,j}$$

$$d_i = \sqrt{\sum_{l=1}^{L}(\bar{x}_{i,l} - \frac{1}{N}\sum_{k=1}^{N}\bar{x}_{k,l})^2 + (\bar{y}_{i,l} - \frac{1}{N}\sum_{k=1}^{N}\bar{y}_{k,l})^2}$$

Where L is the number of landmarks and N the number of images. In order to keep the deformations comparable between the datasets, image heights and widths were set to 1, and the number of landmarks was fixed to $L = 10$. These L landmarks were selected randomly.

The deformation distribution of both approaches is given in Figure 1. From this figure, we can conclude that the deformations in the DROSO dataset are more important than in CEPHA and in ZEBRA.

Figure 2 shows the influence of the importance of the deformation on the error when the distance to the mean shape criterion is used. As it could be expected, RAW, SUB and GAUSSIAN features have more difficulties to handle large deformations than HAAR and SURF features. DMBL also seems to encounter difficulties with bigger deformations. Haar-Like features seems to be the less impacted by the deformations along with LC.

Our algorithm	Tested Values
R	$6, 9, 15, 20$
$R_{\max}$	$100, 300, 500$
P	$1, 3$
N_p	10.000
N_r	$1, 3$
α	$30°$
T	50
D	6
F	RAW,SUB,GAUSSIAN SUB,HAAR-LIKE
DMBL	Tested Values
T (phase 1)	50
F (phase 1)	32
R (phase 1)	$3, 5$
σ	$10, 50, 100, 200$
δ	$0.25, 0.5$
P	$1, 0.5N$
R (phase 2)	$10, 20, 50, 100$
N_s	1000
T (phase 2)	50
F (phase 2)	32
Filter size	$3, 10$
β	$0.2, 0.5$
#Iterations	$1, 3, 5$
#Candidates	1, 5, 10
#Edges	$0.1N, 0.5N, N$
LC	Tested Values
PCA reduc	$1, 25\%, 50\%, 100\%$
$d_{\max}$	$50, 100, 200, 500$
N_s	1000
W	$100, 200, 400$
n	1600
T	50
step	$4, 8, 16$
$R_{\max}$	$100, 300, 500, 1000$
$R_{\min}$	$1, 2, 10, 20$
α	$0.1, 0.5, 0.9$

Table 1: Parameters tested during cross validation on the three datasets for our method, DMBL [6] and LC [10].

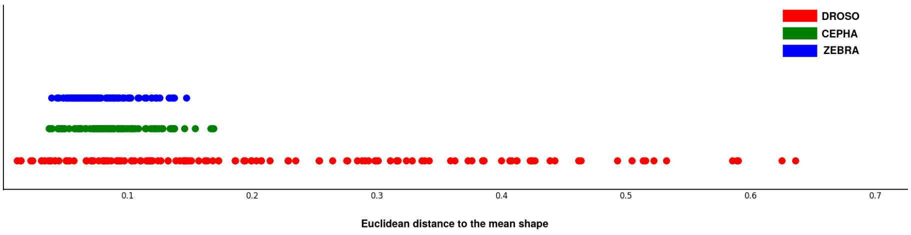

Figure 1: Distribution of the deformation in the images.

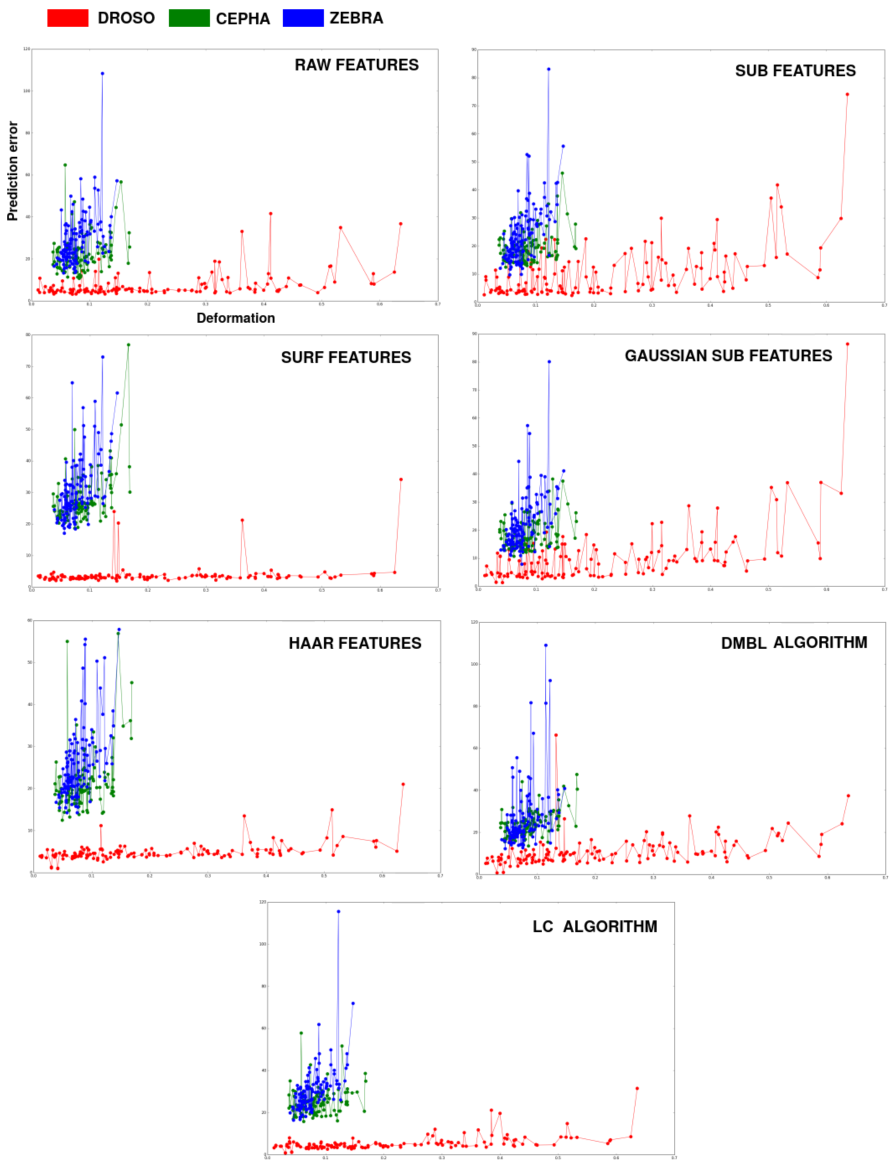

Figure 2: Prediction error according to the mean-based deformation criterion.

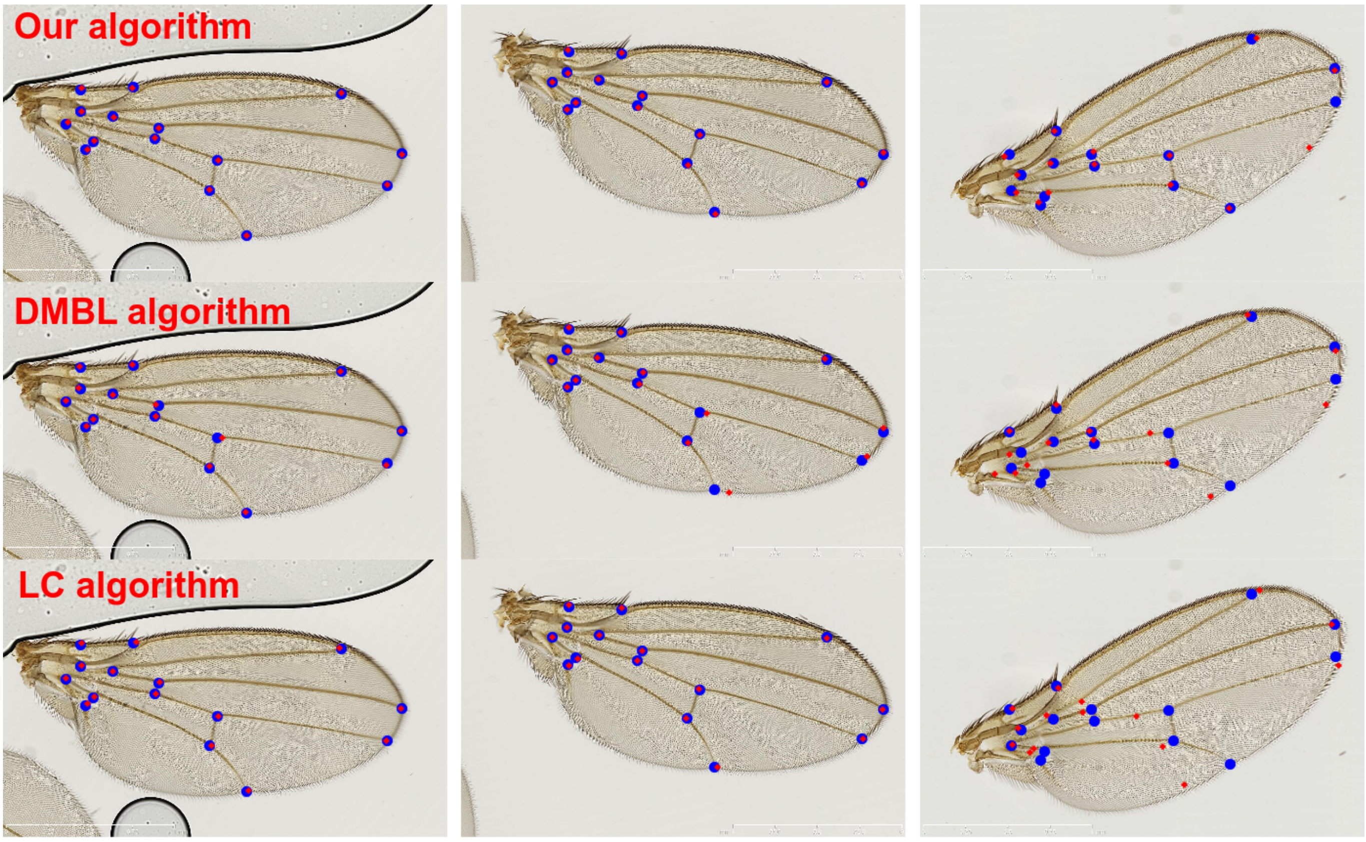

Figure 3: Examples of detection on the DROSO dataset. Ground truth landmarks are in blue, and the detection in red. The 3 images were chosen according to their deformations (smallest, median and maximal deformation)

In Figures 3,4,5, we show examples of detection for our algorithm and the two algorithms we reimplemented [10, 6]. These figures show the detections on images that were according to their deformation. For each dataset, we show the results for the images with the less, median and maximal deformation.

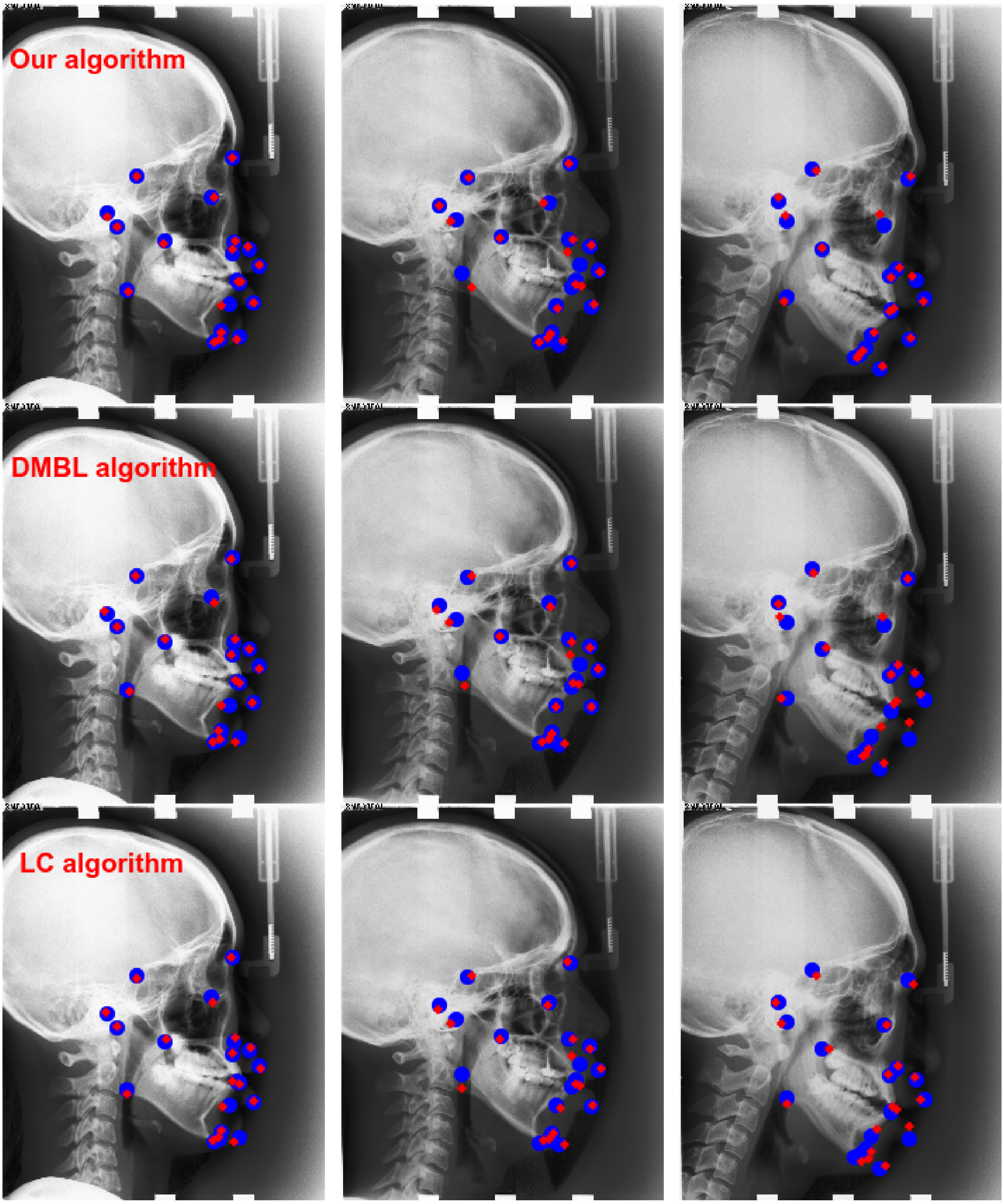

Figure 4: Examples of detection on the CEPHA dataset. Ground truth landmarks are in blue, and the detection in red. The 3 images were chosen according to their deformations (smallest, median and maximal deformation)

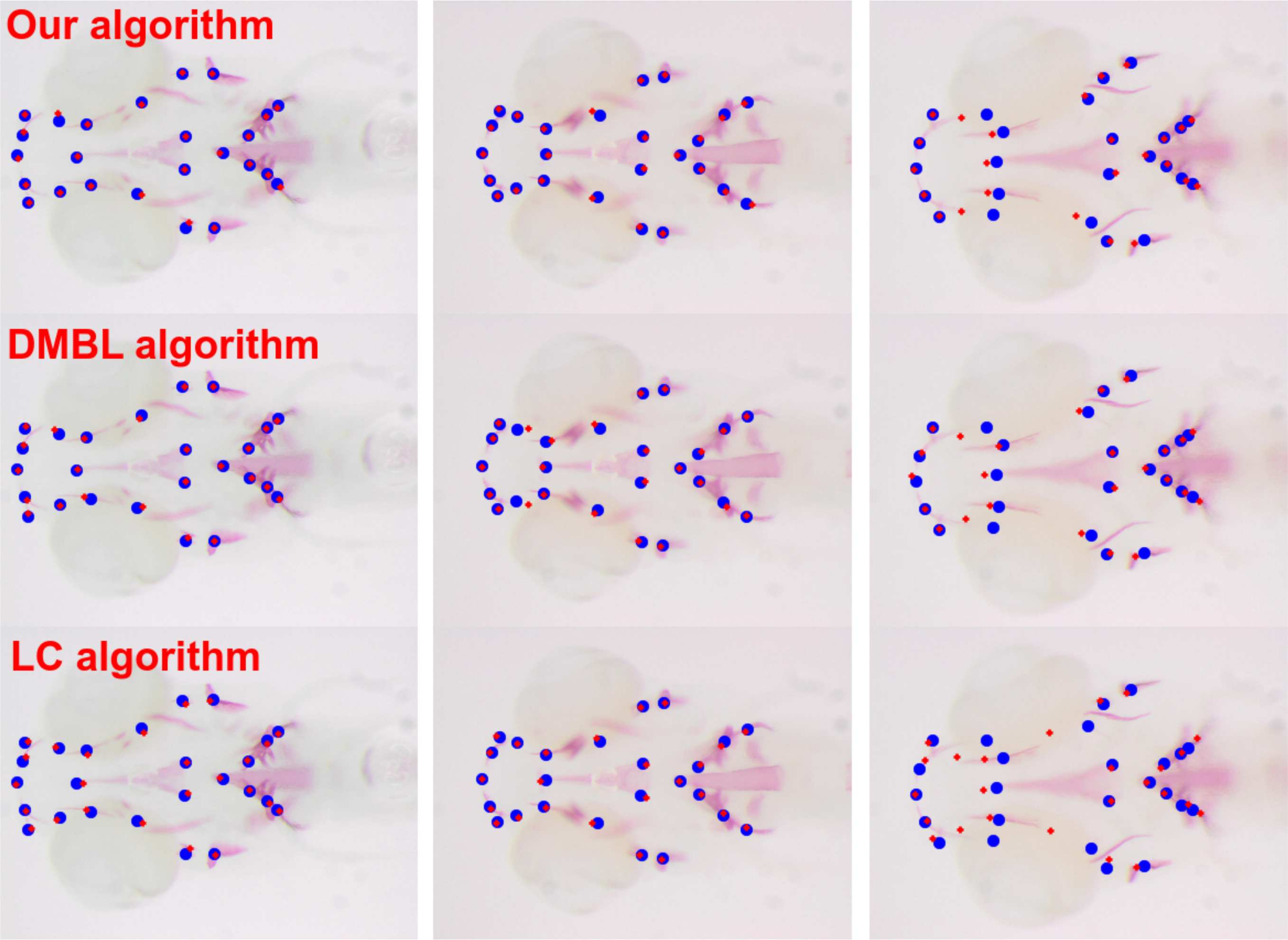

Figure 5: Examples of detection on the ZEBRA dataset. Ground truth landmarks are in blue, and the detection in red. The 3 images were chosen according to their deformations (smallest, median and maximal deformation)

References

[1] Jessica Aceto, Rasoul Nourizadeh-Lillabadi, Raphael Marée, Nadia Dardenne, Nathalie Jeanray, Louis Wehenkel, Peter Aleström, Jack JWA van Loon, and Marc Muller. Zebrafish bone and general physiology are differently affected by hormones or changes in gravity. *PloS one*, 10(6):e0126928, 2015.

[2] Courtney Alexander, Elizabeth Zuniga, Ira L Blitz, Naoyuki Wada, Pierre Le Pabic, Yashar Javidan, Tailin Zhang, Ken W Cho, J Gage Crump, and Thomas F Schilling. Combinatorial roles for bmps and endothelin 1 in patterning the dorsal-ventral axis of the craniofacial skeleton. *Development*, 138(23):5135–5146, 2011.

[3] Alexander Apschner, Stefan Schulte-Merker, and P Eckhard Witten. Not all bones are created equal-using zebrafish and other teleost species in osteogenesis research. *Methods Cell Biol*, 105:239–255, 2011.

[4] Julia Dalcq, Vincent Pasque, Aurélie Ghaye, Arnaud Larbuisson, Patrick Motte, Joseph A Martial, and Marc Muller. Runx3, egr1 and sox9b form a regulatory cascade required to modulate bmp-signaling during cranial cartilage development in zebrafish. *PloS one*, 7(11):e50140, 2012.

[5] Vincent Debat, Sébastien Bloyer, Floria Faradji, Nelly Gidaszewski, Nicolas Navarro, Pablo Orozco-terWengel, Valérie Ribeiro, Christian Schlötterer, Jean S Deutsch, and Frédérique Peronnet. Developmental stability: a major role for cyclin g in drosophila melanogaster. *PLoS Genet*, 7(10):e1002314, 2011.

[6] René Donner, Bjoern H Menze, Horst Bischof, and Georg Langs. Global localization of 3d anatomical structures by pre-filtered hough forests and discrete optimization. *Medical image analysis*, 17(8):1304–1314, 2013.

[7] Charles B Kimmel, Craig T Miller, and Cecilia B Moens. Specification and morphogenesis of the zebrafish larval head skeleton. *Developmental biology*, 233(2):239–257, 2001.

[8] Christian Peter Klingenberg and Stefanie D Zaklan. Morphological integration between developmental compartments in the drosophila wing. *Evolution*, 54(4):1273–1285, 2000.

[9] Arnaud Larbuisson, Julia Dalcq, Joseph A Martial, and Marc Muller. Fgf receptors fgfr1a and fgfr2 control the function of pharyngeal endoderm in late cranial cartilage development. *Differentiation*, 86(4):192–206, 2013.

[10] Claudia Lindner and Tim F Cootes. Fully automatic cephalometric evaluation using random forest regression-voting. 2015.

[11] Eirinn W Mackay, Alexander Apschner, and Stefan Schulte-Merker. A bone to pick with zebrafish. *BoneKEy reports*, 2, 2013.

[12] R. Marée, L. Rollus, B. Stévens, R. Hoyoux, G. Louppe, R. Vandaele, J.-M. Begon, P. Kainz, P. Geurts, and L. Wehenkel. Collaborative analysis of multi-gigapixel imaging data using cytomine. *Bioinformatics*, 2016.

[13] Thomas F Schilling, Tatjana Piotrowski, Heiner Grandel, Michael Brand, Carl-Philipp Heisenberg, Yun-Jin Jiang, Dirk Beuchle, Matthias Hammerschmidt, Donald A Kane, Mary C Mullins, et al. Jaw and branchial arch mutants in zebrafish i: branchial arches. *Development*, 123(1):329–344, 1996.

[14] Ching-Wei Wang, Cheng-Ta Huang, Jia-Hong Lee, Chung-Hsing Li, Sheng-Wei Chang, Ming-Jhih Siao, Tat-Ming Lai, Bulat Ibragimov, Tomaž Vrtovec, Olaf Ronneberger, Philipp Fischer, Tim F. Cootes, and Claudia Lindner. A benchmark for comparison of dental radiography analysis algorithms. *Medical Image Analysis*, 2016.

[15] CW Wang, CT Huang, MC Hsieh, CH Li, SW Chang, WC Li, R Vandaele, R Maree, S Jodogne, P Geurts, C Chen, G Zheng, Chu CW, Mirzaalian H, Hamarneh G, Vrtovec T, and Ibragimov B. Evaluation and comparison of anatomical landmark detection methods for cephalometric x-ray images: A grand challenge. *IEEE TMI*, 34(9):1890–1900, 2015.

[16] Monte Westerfield. *The zebrafish book: a guide for the laboratory use of zebrafish (Danio rerio).* University of Oregon press, 2000.

[17] Thomas Windhausen, Steeve Squifflet, Jörg Renn, and Marc Muller. Bmp signaling regulates bone morphogenesis in zebrafish through promoting osteoblast function as assessed by their nitric oxide production. *Molecules*, 20(5):7586–7601, 2015.
